# Combined petrosal approach for resection of a large left petroclival meningioma

**DOI:** 10.3171/2022.1.FOCVID21226

**Published:** 2022-04-01

**Authors:** Francesco Paglia, Lorenzo Giammattei, Paolo di Russo, Sebastien Froelich

**Affiliations:** Department of Neurosurgery, Lariboisiere Hospital, Paris Diderot University, Paris, France

**Keywords:** combined petrosal approach, petroclival meningioma, tentorial cutting, cosmetic mastoidectomy, transverse-sigmoid junction, Labbé vein, superior petrosal sinus, semicircular canals

## Abstract

Petroclival meningiomas represent the most complex lesions in skull base surgery, being closely related to critical neurovascular structures. The combined petrosal approach allows a wide exposure of the petroclival region and provides multiple angles of attack, limiting brain retraction.

The authors present the case of a 54-year-old man with a large left petroclival meningioma responsible for headaches, dysphagia, and trigeminal neuralgia. The lesion was resected using a combined petrosal approach. A progressive improvement of the preoperative symptoms was observed. Postoperative MRI showed a near-total resection of the tumor, along with reexpansion of the brainstem.

The video can be found here: https://stream.cadmore.media/r10.3171/2022.1.FOCVID21226

## Transcript

This video shows a combined petrosal approach for resection of a petroclival meningioma.

### 0:25 Clinical Presentation and Neurological Examination.

The patient was admitted for headache, dysphagia, and left facial pain.

### 0:29 Neuroimaging.

The MRI showed a left petroclival meningioma that progressively increased on radiological follow-up and was associated with worsening of neurological symptoms.

### 0:39 Choice of the Approach.

The combined petrosal approach was chosen for different reasons: firstly, the inferior limit of the tumor was lower than the internal acoustic meatus. Secondly, anterior petrosal approach does not provide an optimal control over the seventh and eighth cranial nerves in their cisternal segment. Thirdly, the combined petrosal with posterior transposition of sigmoid sinus offers an upward line of sight, which provided adequate exposure of the superior aspect of the tumor, third cranial nerve, and posterior communicating artery. Finally, this approach provides multiple lines of sight, each of which can be used for optimal dissection of the various cranial nerves and major vessels.

### 1:18 Preoperative Audiogram.

The audiogram showed a serviceable hearing; for this reason, a retrolabyrinthine petrosectomy was preferred.

### 1:25 Preoperative Radiological Considerations.

MRI did not show any brainstem edema.

The vein of Labbè presents a regular anatomical conformation. Preoperative CT scan is helpful in the assessment of the bony anatomy, including tumor’s calcifications, pneumatization of the petrous apex, and the retromeatal space. Is also important to evaluate a possible dehiscence of the middle fossa floor over the geniculate ganglion, as well as a dehiscence at the level of the carotid canal in order to reduce the risk of injury of these structures during the approach.

Finally, we evaluate also the height of the jugular bulb in relation to the internal acoustic canal.

### 2:07 Preoperative Angiography and Embolization.

Preoperative angiography allows to evaluate the vascularization of the tumor. This sequence shows a blush coming from the ascending pharyngeal artery and also from the dural branches of the internal carotid artery, represented by the inferolateral trunk and meningohypophyseal trunk. Embolization, through coils at the level of the ascending pharyngeal artery, performed the day before the surgery, enables to reduce significantly the intraoperative bleeding.

### 2:31 Patient’s Positioning.

The patient is put in supine position with the head fixed in a three-pins skull’s clamp and rotated 70° to 80° to the contralateral side.

### 2:39 Skin Incision.

A C-shaped skin incision is made starting from the tip of mastoid and encircling the approximate location of the temporalis muscle.

### 2:48 Necessary Equipment.

Cranial nerves and somatosensory and motor evoked potentials are monitored. Neuronavigation is used to locate the transverse-sigmoid junction.

### 2:58 Interfascial Dissection of the Temporalis Muscle.

The first surgical step is represented by the interfascial dissection of the temporalis muscle in order to release the skin flap. Once the fat pad is found, an interfascial dissection is performed and then the temporal muscle is elevated in a retrograde fashion subperiosteally.

### 3:20 Nuchal Muscle Detachment.

The monopolar is used to detach the sternocleidomastoid muscle from the superior nuchal line. Digastric muscle is also detached. At the end of this procedure, these muscles are retracted inferiorly. The posterior margin of the external acoustic canal is progressively found and also the root of the zygoma.

### 3:38 Craniotomy.

A burr hole is performed at the level of the transverse-sigmoid junction, and position of the sinus is confirmed by intraoperative Doppler. Another burr hole is anteriorly performed at the level of the temporal bone, and then a one-piece temporal and retrosigmoid craniotomy is performed.

### 3:53 Cosmetic Mastoidectomy.

Using a craniotome without footplate and a bone scalpel, a cosmetic mastoidectomy is progressively obtained.

### 4:01 Posterior Petrosectomy.

Using cutting and diamond burrs, mastoidectomy is performed with progressive identification of the sinodural angle and labyrinthine block. Skeletonization of the sigmoid sinus is done by diamond burr and rongeur.

### 4:16 Anterior Petrosectomy.

Middle fossa is flattened, and then we progressively identify foramen spinosum.

Middle meningeal artery is coagulated and cut. Peeling of the dura is performed with progressive identification of GSPN, V3, arcuate eminence, and petrous ridge that are the limits of the Kawase rhomboid.

In case of a tense brain, the retrosigmoid dura is opened to access the lower cranial nerves’ cistern and release CSF. The drilling of the Kawase rhomboid should start in the safest region, which is next to V3, where a hole is created. Then this cavity is progressively enlarged in a posterior and posterolateral direction under constant irrigation until the dura of the posterior fossa is exposed.

The anterior petrosectomy is then extended inferiorly and posteriorly till reaching cortical ivory bone surrounding the cochlea and the superior semicircular canals.

### 5:16 Temporal Dura Incision.

The temporal dura is incised. Another incision is made more laterally and posteriorly to identify the vein of Labbé and avoid its injury. Subsequently, the dura is used to retract the sinus. Patency of eventual tentorial venous channels is verified using intraoperative Doppler.

### 5:38 Presigmoid Dura Incision.

Presigmoid dura is progressively opened in a semicircular fashion running anteriorly below the SPS.

### 5:47 Tentorial Detachment.

Tentorium resection starts with a posterior cut toward the free edge.

Then an anterior cut is performed starting at the level of the porus trigeminalis. The dura is very thick and infiltrated by the tumor. A piece of infiltrated tentorium is then progressively removed with a Kerrison punch in a piecemeal fashion, allowing a wide exposure of the superior aspect of the tumor.

Then the dura of the lateral aspect of the porus trigeminalis and Meckel’s cave is also opened, exposing V3.

### 6:34 Cranial Nerve Dissection From the Tumor.

The two-forceps technique is used to preserve the arachnoidal plane around neurovascular structures. The fourth cranial nerve is visualized under the tentorium. Ultrasonic aspirator is used to perform a progressive debulking of the tumor, alternated to gentle bimanual dissection.

This technique is mandatory to preserve basilar artery perforators located in this delicate area.

### 7:05 Dissection of the Perforators From the Lesion.

Debulking of the tumor alternates with the two-forceps technique. After identification of the basilar artery, low aspiration and microscissors are utilized to progressively mobilize the tumor and dissect it freely from these vessels. This delicate procedure is done under high magnification in order to cut safely the small arachnoidal bridges connecting the tumor to the basilar artery and its perforators.

The dorsal surface of the tumor is also dissected and mobilized using the same technique. In case of brainstem edema and tight adherence to the brainstem, remnants of the tumor should be left behind to avoid any injury of these perforators.

### 7:48 Identification of Third Cranial Nerve.

Progressively, the third cranial nerve is identified and dissected free from the tumor and also the sixth cranial nerve going toward Dorello’s canal.

### 7:58 Closure.

The temporal dura is then reapproximated with 5-0 dural stitches. Then a thin layer of bone wax was used to seal the middle ear. Pericranium is applied over the tegmen tympani and fixed with some glue. Abundant abdominal fat tissue is positioned into the surgical cavity in order to fill the dead space created by the combined petrosal approach. Fibrin glue is used in order to secure the fat. A continuous running suture is performed over the temporal and retrosigmoid dura in order to obtain a satisfying dural closure. The mastoid cortical bone and the bone flap were replaced and attached with miniplates.

### 8:43 Postoperative Neuroimaging.

Postoperative CT scan does not show any postoperative complication. Postoperative MRI shows the near-total resection of the meningioma with a small residuum in the posterior part of the cavernous sinus.

### 8:57 Postoperative Clinical Outcome.

During the postoperative course, the patient presented a partial fourth cranial nerve palsy with a slight diplopia when looking down that recovered completely after 3 months and a slight hearing decrease due to otitis media, which was completely resolved after 6 months.

### 9:13 References^1–6^

## Disclosures

The authors report no conflict of interest concerning the materials or methods used in this study or the findings specified in this publication.

## Author Contributions

Primary surgeon: Froelich. Assistant surgeon: Paglia, Giammattei, di Russo. Editing and drafting the video and abstract: Paglia, Giammattei, di Russo. Critically revising the work: Paglia, Giammattei, Froelich. Reviewed submitted version of the work: Paglia, Giammattei, Froelich. Approved the final version of the work on behalf of all authors: Paglia. Supervision: Froelich.

## References

[1] Hanakita S Watanabe K Champagne PO Froelich S How I do it: combined petrosectomy Acta Neurochir (Wien) 2019 161 11 2343 2347 31410555 10.1007/s00701-019-04022-z

[2] Fournier HD Mercier P Roche PH Surgical anatomy of the petrous apex and petroclival region Adv Tech Stand Neurosurg. 2007 32 91 146 10.1007/978-3-211-47423-5_5 17907476

[3] Abdel Aziz KM Sanan A van Loveren HR Tew JM Jr Keller JT Pensak ML Petroclival meningiomas: predictive parameters for transpetrosal approaches Neurosurgery 2000 47 1 139 152 10917357

[4] Al-Mefty O Fox JL Smith RR Petrosal approach for petroclival meningiomas Neurosurgery 1988 22 3 510 517 3362317 10.1227/00006123-198803000-00010

[5] Hakuba A Nishimura S Jang BJ A combined retroauricular and preauricular transpetrosal-transtentorial approach to clivus meningiomas Surg Neurol 1988 30 2 108 116 3400039 10.1016/0090-3019(88)90095-x

[6] Haq IB Susilo RI Goto T Ohata K Dural incision in the petrosal approach with preservation of the superior petrosal vein J Neurosurg 2016 124 4 1074 1078 26339848 10.3171/2015.3.JNS141618

